# Encapsulation of Benzyl Isothiocyanate with β-Cyclodextrin Using Ultrasonication: Preparation, Characterization, and Antibacterial Assay

**DOI:** 10.3390/foods11223724

**Published:** 2022-11-20

**Authors:** Hongbo Li, Xujia Ming, Zhen Wang, Jiaqi Li, Yunxia Liang, Dan Xu, Zhenbin Liu, Liangbin Hu, Haizhen Mo

**Affiliations:** 1 School of Food Science and Engineering, Shaanxi University of Science and Technology, Xi’an 710021, China; 2 School of Chemistry and Chemical Engineering, Ocean University of China, Qingdao 266000, China; 3 Department of Food Science, Henan Institute of Science and Technology, Xinxiang 453003, China

**Keywords:** benzyl isothiocyanate, BITC-β-CD complex, antimicrobial activity, broccoli juice

## Abstract

Benzyl isothiocyanate (BITC) is widely utilized in multiple biomedical fields, due to its significant antibacterial properties and low toxicity. However, poor water solubility and pungent odor has limited its application in the food industry. In this study, we first prepared inclusion complexes of BITC in GLU-β-CD and HP-β-CD using ultrasound, which is able to overcome the hindrance of poor water solubility and high volatility. Then, the BITC-β-CD inclusion complexes were characterized by using high-performance liquid chromatography (HPLC), nuclear magnetic resonance hydrogen spectra (^1^H-NMR), infrared absorption spectra (IR), and differential scanning calorimetry (DSC) to confirm their stability. Further, the evaluation of antibacterial and antitumor effects of the BITC-β-CD inclusion complexes showed that they had great bactericidal activity against both *Escherichia coli* and *Staphylococcus aureus* cells, and also inhibited the growth of HepG2 cells in vitro. In addition, our results indicated that BITC-β-CD complexes were able to inhibit the growth of *S. aureus* in broccoli juice and extend the shelf life of broccoli juice, demonstrating the potential of β-cyclodextrin to improve the stability and controlled release of BITC. Taken together, our results show that BITC-β-CD complexes have good potential for application in the food industry.

## 1. Introduction

It is well known that microbial contamination is a threat to both food safety and quality. Foodborne pathogens are one of the most commonly reported causes of food contamination outbreaks, especially in fresh fruits and vegetables [1]. The current mainstream treatment of foodborne pathogens is still predominantly antibiotics. However, antibiotic resistance has become a major public health concern in the 21st century, and infections by antibiotic-resistant microbes are projected to cause 10 million deaths annually by 2050 [2]. Thus, it is urgent to explore new antibacterial agents to combat this crisis. Many essential oils obtained from cruciferous plants have been proven to have antimicrobial activity and are suitable for applications in the food industry. Isothiocyanates (ITCs) are sulfur-containing compounds present in large amounts in cruciferous vegetables, such as broccoli, cabbage, and kale [3], and exhibit broad-spectrum antibacterial activity [4]. Benzyl isothiocyanate (BITC) neutralizes the effects of chemical carcinogenesis in different preclinical cancer models, and is highly effective against both sensitive and resistant bacteria [3,5,6]. Despite its excellent biological properties, the applications of BITC in the food industry are very limited due to its poor water solubility, volatility, instability, photosensitivity, and irritant odor [7].

Cyclodextrins (CDs) and their derivatives are widely used as additives in food processing to enhance aqueous solubility of poorly soluble ingredients, low stability, and poor bioavailability [8,9]. Orally ingested β-CDs and their derivatives are primarily digested by bacteria in the colon, whereas parenterally administered β-CDs are largely excreted in their unmetabolized forms through the urine, and a small amount is metabolized in the liver and eliminated via biliary excretion [10]. Although β-CDs and their hydrophilic derivatives are practically non-toxic [11], the low solubility of natural β-CDs (18.5 mg/mL at 25 °C) in water limits their application as solubilizing and complexing agents. Conversion of natural CDs to a mixture of isomers not only increases the solubility of the CDs but also that of their complexes [7,12]. Our aim in the present study was to enhance the solubility of BITC by encapsulating it in β-CD derivatives, thus improving the antibacterial effect on *Staphylococcus aureus*. The characterizations of BITC/β-CDs were investigated by FT-IR, DSC, particle size distribution analysis, and 1H nuclear magnetic resonance analysis. The results confirmed that BITC was encapsulated in cyclodextrin. The antibacterial activity of BITC-β-CD complex and its application prospect in broccoli juice were also evaluated.

## 2. Materials and Methods

### 2.1. Plant Materials and Chemicals

#### Materials

BITC (98%, MW: 149.2129) was purchased from Adamas Reagent Ltd. (Shanghai, China). HP-β-CD (98%, MW: 1540) and GLU-β-CD (98%, MW: 1297) were purchased from Shanghai Yuanye Bio-Technology (Shanghai, China). Other chemicals were all of analytical grade.

### 2.2. Molecular Modeling

The 3D structure of BITC was constructed using Gaussian 09. The structural and bioenergetic properties of BITC-β-CDs were analyzed by semiempirical quantum mechanical calculation at PM3 level of theory [13]. The 3D structure of β-CDs was obtained from the RCSB Protein Data Bank (PDB ID: 4YEF) [14] and modified to obtain HP-β-CD and GLU-β-CD pymol. The molecular docking of BITC into HP-β-CD and GLU-β-CD were simulated using the Autodock 4.2 software to obtain the most stable possible structure.

### 2.3. Solubility of BITC in β-CD Derivatives

Phase solubility studies were performed according to standard procedures [15]. The inclusion complex of BITC with HP-β-CD and GLU-β-CD were prepared as described by Li et al. with minor modifications [16]. Briefly, a series of β-CD derivatives of concentrations 0, 0.1, 0.2,0.3, 0.4, 0.5, and 0.6 mM were prepared in a 40 °C water bath. BITC solution (0.2 μL) was slowly blended into 1 mL of the prepared β-CD derivative solution. Subsequently, the mixed solution was treated with sonication (power 100%) for 3 min at intervals of 1 s per pulse and shaken until a clear solution was obtained. The final volume of BITC was recorded [17,18], and the resulting solution was frozen at −80 °C. Afterwards, the suspensions were filtered through 0.45 μm hydrophilic cellulose membrane filters to remove undissolved BITC. The concentration of BITC in the filtrate from each tube was determined by high-performance liquid chromatography (HPLC). All experiments were run in triplicates. The phase solubility profiles of BITC were obtained by plotting the solubility of BITC vs. the concentration of the β-CDs. The apparent stability constants (Ks) of BITC–β-CD complexes were calculated from phase-solubility diagrams according to the following equation: Ks = slope/so(1-slope), where so is the solubility of BITC in the absence of β-CDs, and slope means the corresponding slope of the phase solubility diagram.

### 2.4. Preparation of BITC Inclusion Complexes

The inclusion complex was prepared with 1 g/mL β-cyclodextrin derivative solution according to the results of the solubility experiments. The preparation process of the inclusion complex was based on the solubility experiment. BITC solution (1 µL) was slowly blended into 1 mL of the prepared β-CD derivative solution. The inclusion solution was frozen at −20 °C for 12 h and freeze-dried for 24 h until BITC-β-CD-lyophilized powders were obtained. The final volumes of the BITC-β-CD-lyophilized powders were recorded, and the resulting lyophilized powders were stored at 4 °C. 

### 2.5. Determination of Inclusion Rate

A high-performance liquid chromatography (HPLC1260, Agilent Technologies, Santa Clara, CA, USA) assay was used to determine standard BITC concentration curves. Different concentrations of BITC were dissolved in 1 mL of methanol to final concentrations of 240, 120, 60, 30, 15, 7.5, 3.75, 1.875, 0.94, 0.47, 0.235, and 0.1175 μM/mL, and filtered through 0.22µm hydrophilic membrane, and then 10 µL of filtrate was injected into the C18 column (4.6 mm × 250 mm, 5 µm). Chromatographic separation was performed at 40 °C using a mobile phase of deionized water/acetonitrile (40:60, *v*/*v*) at a flow rate of 1 mL/min. The detection wavelength was 268 nm. 

Following chromatographic separation, 100 milligrams of each BITC-β-CD-lyophilized powder was added to 1 mL of methanol solution. The solutions were filtered through a membrane with mean pore size of 0.22 µm, and the concentration of BITC was determined by high-performance liquid chromatography (HPLC).

The inclusion rate (IR) was calculated, as follows [17]:

(1) $$\mathrm{IR}\%=\frac{\mathrm{amount}\;\mathrm{of}\;\mathrm{BITC}\;\mathrm{entrapped}}{\mathrm{amount}\;\mathrm{of}\;\mathrm{initial}\;\mathrm{BITC}\;\mathrm{dropped}}\times 100\%$$

### 2.6. Determination of Inclusion Stability

The freeze-dried powder was sealed and dried for 10, 20, 30, and 40 days. The remaining BITC content was measured, and the determination method was the same as that for HPLC. The inclusion stability (IS) was calculated using the following equation [9]:

(2) $$\mathrm{IS}\%=\frac{\mathrm{amount}\;\mathrm{of}\;\mathrm{BITC}\;\mathrm{content}\;\mathrm{after}\;\mathrm{storage}}{\mathrm{amount}\;\mathrm{of}\;\mathrm{BITC}\;\mathrm{entrapped}}\times 100\%$$

### 2.7. Particle Size Distribution

The average droplet size of β-CD emulsions and their polydispersity index (PDI) were measured using the Malvern Zetasizer Nano-ZS90 (Malvern, UK). Aqueous solutions of HP-β-CD and GLU-β-CD were prepared at the concentration of 0.1 g/mL. In addition, 1 µL (37.5 µM) BITC was dissolved in 1 mL HP-β-CD and GLU-β-CD, and uniformly mixed with ultrasonic vibration. The Z-average size value was calculated by the methods of cumulants [19]. 

### 2.8. Infrared Spectroscopy (IR)

GLU-β-CD, HP-β-CD, BITC, and corresponding inclusion complexes were respectively powdered with dry KBr (1:100) to obtain transparent tablets for analyses. Infrared spectroscopy (TENSOR 27, Bruker, Bremen, Germany) of the different samples was performed within the wavelength range of 400 to 4000 cm^−1^. OPUS software was used to collect and analyze data.

### 2.9. Differential Scanning Calorimetry (DSC)

The heat stability of the GLU-β-CD, HP-β-CD, and BITC inclusion complexes was evaluated by differential scanning calorimetry (DSC Q200, TA, USA). The lyophilized powders (1 mg) were heated in a hermetically sealed aluminum disk from 25 to 300 °C at the rate of 10 °C/min. The flow rate of N2 was 50 mL/min [20].

### 2.10. ^1^H Nuclear Magnetic Resonance (^1^H-NMR)

The freeze-dried HP-β-CD, GLU-β-CD, and BITC inclusion complex powders were dissolved in D2O and equilibrated at room temperature for 72 h. The formation of β-cyclodextrin derivatives and BITC-cyclodextrin complex was analyzed on a 400 MHz Bruker Avance spectrometer (AV-400, Bruker, Fällanden, Switzerland) at 25 °C. Chemical shift (δ) was expressed as parts per million (ppm) by frequency using HOD signal as the reference [21].

### 2.11. Antibacterial Activities

*S. aureus* ATCC25923 was cultured in tryptic soy broth (TSB) and *E. coli* MG1655 was grown in Luria–Bertani broth (LB) overnight at 37 °C with shaking. The minimum inhibitory concentration (MIC) of BITC and BITC inclusion complexes on *S. aureus* and *E. coli* were determined using the broth microdilution method according to the Clinical Laboratory Standardization Institute standards [22]. Following MIC determination, 190 µL of (≈10^6^ CFU/mL) *S. aureus* cells and *E. coli* cells were added into the wells of 96-well microtiter plates (round hole, flat bottom), respectively. Then, BITC and BITC inclusion complexes (10 µL) were added to each well to final concentrations of 2.5, 5, 10, 20, 40, 80, and 160 µg/mL, respectively. The 96-well plates were incubated at 37 °C, 200 rpm/min for 12 h. Bacterial growth was evaluated by measuring optical density at 600 nm (OD600) using a microreader (G9800A, Thermol, CA, USA) [23]. The MIC was defined as the lowest concentration of BITC and BITC inclusion complexes that inhibited visible growth. The drop plate method was used to compare the inhibitory activity of BITC and BITC inclusion complexes to *S. aureus* and *E. coli* strains. Briefly, *S. aureus* and *E. coli* were diluted to different concentrations (10^−1^, 10^−2^, 10^−3^, 10^−4^, and 10^−5^) after being cultured to OD600 = 0.8. Plates were coated by treating *S. aureus* and *E. coli* with BITC and BITC inclusion compounds at a concentration of 40 µg/mL. The minimal bactericidal concentration (MBC) was determined by spreading the culture from each well onto nutrient agar plates and measuring no growth after incubation at 37 °C for 24 h.

### 2.12. Antitumor Activities

Human hepatocellular carcinoma (HepG2) cells were obtained from Xinxiang Medical University. The cancer cells were routinely cultured in RPMI-1640 medium supplemented with 10% fetal bovine serum under weakly alkaline conditions (pH: 7.2–7.4). These cells were placed at 37 °C with 5% CO_2_ purification until used.

The cancer cell suspension in logarithmic growth phase was inoculated in 96-well plates (cell density was adjusted to 1 × 10^4^ cells per well) and exposed to different concentrations of BITC and BITC inclusion complexes (1, 2, 4, 8, 16, 32, 64, and 128 μg/mL). The control group was pure culture solution. The cells were cultured in a humid atmosphere of 37 °C and 5% CO_2_ [24]. After 24 h, 10 μL of MTT and 90 μL of fresh culture medium were replaced and the cells were cultured for another 4 h. After incubation, 100 μL DMSO was added to each well. The plate was slowly shaken at room temperature for 10 min. Then the absorbance of the DMSO–cell solution was detected by ELISA Reader at 490 nm [25]. The detection was performed in parallel 3 times, and the average value was used to calculate the cell viability. 

### 2.13. Antimicrobial Activity during Broccoli Juice Pasteurization

Broccoli was purchased from a local market. The fresh broccoli was disinfected, washed, peeled, and squeezed to obtain the juice. The broccoli juice (100 mL) was dispensed in sterilized conical flasks (250 mL) under aseptic conditions. Four treatments were tested on broccoli juice: (1) control juice, (2) juice + free BITC (40 μg/mL), (3) juice + BITC encapsulated in HP-β-CD (1 g/mL), and (4) juice + BITC encapsulated in GLU-β-CD (1 g/mL). The juice samples were mixed with TSB, which was then inoculated with *S. aureus* cells of 10^6^ CFU/mL. The inoculated samples were stored at 4 °C for 8 days, and the number of live bacterial cells were counted on days 0, 2, 4, and 8. Briefly, 100 µL of each sample (in triplicate) was diluted with 0.85% sterile saline and plated on TSB agar and incubated at 37 °C. The total viable count was determined by the plate colony counting method as log CFU/mL.

### 2.14. Statistical Analysis

All experiments were performed in triplicate. Data was expressed as the mean ± standard deviation (SD). Statistical analysis was performed using Student’s *t*-test, and a *p* value of <0.05 was considered statistically significant.

## 3. Results and Discussion

### 3.1. Molecular Modeling

BITC is a nutritional component of cruciferous origin with excellent antitumor activity and antimicrobial ability to control foodborne pathogen proliferation. Soundararajan et al. demonstrated that BITC, PEITC, and SFN targeted proteins associated with cell proliferation and homeostasis, interacting with proteins involved in the repair of DNA damage, thereby stimulating cell cycle arrest and induction of programmed cell death [26]. Lee et al. treated oral cancer CAR cells with different concentrations of BITC for 24 and 48 h, and the results of MTT assay showed that BITC inhibited the growth of CAR cells in a concentration-dependent manner [27]. However, BITCs always show poor stability in most biological media due to their evidently electrophilic chemical function. Additionally, the high volatility of BITC can weaken its antibacterial effect against pathogens and hinder its potential use as an effective bioactive agent. An effective means to solve this problem is to identify the appropriate carriers to completely attain or at least improve the stability of BITC cyclodextrin (CD) as a plausible carrier for various essential oil compounds. Research showed that encapsulation of BITC with CD and its derivatives is helpful in inhibiting the decomposition of BITC in aqueous solution [28,29,30,31]. Therefore, the long-term controlled release of BITC by encapsulation of BITC with CD will be of great significance to improve its inhibitory effect on pathogens. Liu et al. developed inclusions consisting of γ-cyclodextrin (γ-CD) and benzyl isothiocyanate (BITC), and phenethyl isothiocyanate (PEITC) and 3-methylthiopropyl isothiocyanate (MTPITC), and quantitative crystalline violet analysis and scanning electron microscopy results showed that *S. aureus* treated with γ-CD-BITC formed less biofilm than BITC-treated *S. aureus* [31]. An α-CD encapsulation of moringa isothiocyanate promoted aqueous solubility and stability, which in turn exhibited a higher efficacy in suppressing neuroblastoma cell proliferation than solely applying the BITC compound [32]. However, the small cavity structure of α-CD limited its encapsulation of some medium and large molecules.

**Figure 1:**
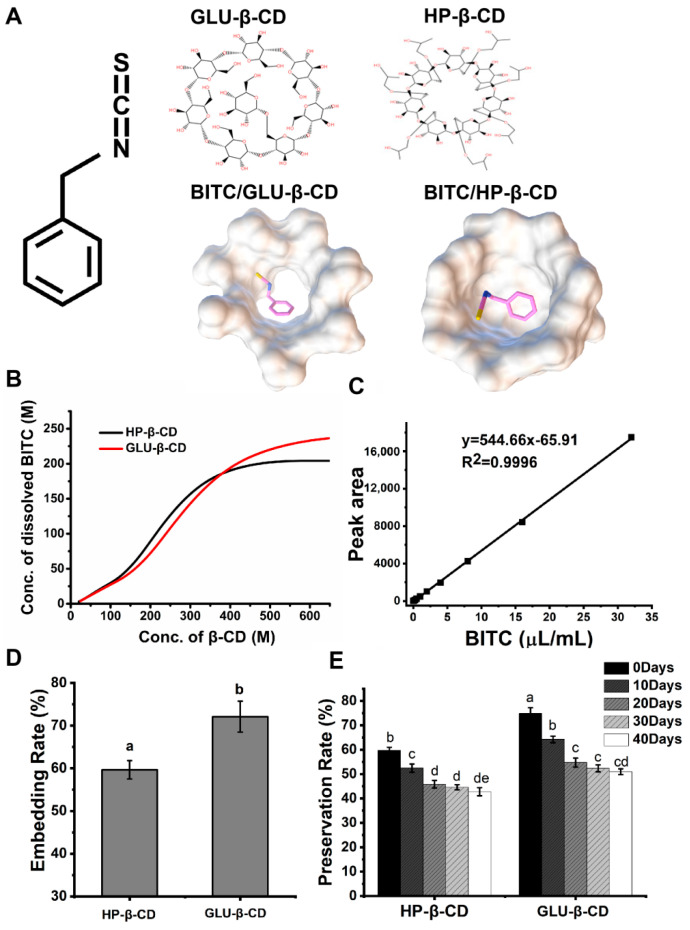
(**A**) Molecular simulated conformation of BITC-β-CD complexes. (**B**) Dissolved phase of BITC in the β-CD derivatives. (**C**) Standard curve of BITC. (**D**) Embedding rate of BITC in β-CD derivatives. (**E**) Stabilization of β-CD derivatives with BITC. Letters a-e represent statistically significant values at *p* ≤ 0.05.

In the present study, we prepared BITC-β-CD complexes using ultrasonic encapsulation of benzyl isothiocyanate with β-cyclodextrin, and molecular modeling was used to visualize the molecular interactions and spatial orientation in inclusion complexes [33]. The binding conformation and energy of BITC-β-CD complexes were analyzed using Autodock 4.2. The most stable and smallest energy structures between BITC and HP-β-CD/GLU-β-CD are shown in Figure 1A. BITC was embedded in the hydrophobic cavity of the β-CD derivatives through hydrophobic interactions rather than hydrogen bonding and chemical bonding. Thus, molecular docking results showed that BITC could enter the cavities of β-CD derivatives to form clathrate compounds [34].

### 3.2. Solubilization of BITC

The stoichiometric characteristics of the complexes and the effects of HP-β-CD and GLU-β-CD complexes on the solubility of BITC were investigated by phase solubility analysis. The phase solubility curves of BITC with HP-β-CD and GLU-β-CD are shown in Figure 1B. The solubility of BITC was significantly improved after inclusion with β-CDs. With the increase of HP-β-CD and GLU-β-CD concentrations, the solubility of the drug increased nonlinearly, which was due to the change in the stoichiometry of the BITC-β-CD derivative complex. The phase solubility curve of BITC in β-CDs was that of A_N_ type [9], with a regression equation of [peak area] = 544.66 [BITC] − 65.91 (determination coefficient, R^2^ = 0.9996), indicating that the ratio of BITC to CDs in the complex was less than 1:1 (Figure 1C). Referring to the research results of Higuchi and Connors, the non-linearity of the A_N_ type phase solubility curve distribution and the negative deviation from normal linearity might be caused by changes in the aqueous medium. Therefore, both cyclodextrins were used at a concentration of 1 g/mL for subsequent experimental studies in order to ensure better experimental conditions. Encapsulation efficiency is an indicator to measure the encapsulation efficiency of bioactive compounds, which in turn determines their application potential [26].

### 3.3. Embedding Rate and Preservation Rate of BITC

BITC embedding rates of two β-CDs derivative inclusion complexes were determined by HPLC at different storage periods (Figure 1D). The results showed that the embedding rate of BITC in GLU-β-CD was 12.44% higher than that in HP-β-CD. This could be attributed to the difference in substituent constant and the molar refractivity between HP-β-CD and GLU-β-CD [29]. The presence of glucosyl replaced oxhydryl in the hydrophobic cavity of GLU-β-CD and facilitated the entry of BITC. HP-β-CD was obtained by substitution of the hydroxyl groups on the exterior surface of β-CD with hydroxypropyl groups. Therefore, BITC could not easily enter or exit the HP-β-CD cavity. While this lowered the inclusion rate into HP-β-CD compared to that for GLU-β-CD, it enhanced the preservation rate of the former. As shown in Figure 1E, the embedded BITC was gradually released and volatilized from CD, and the volatilization rate decreased with prolonged storage. The reason might involve the fact that that the loosely bound BITC molecules were rapidly released in the initial stages, and the tightly bound molecules were released more slowly over a period of 20 days [19]. The results indicated that encapsulation of BITC into the CD complexes slowed its release and prolonged stable storage.

### 3.4. Particle Size Analysis

**Figure 2:**
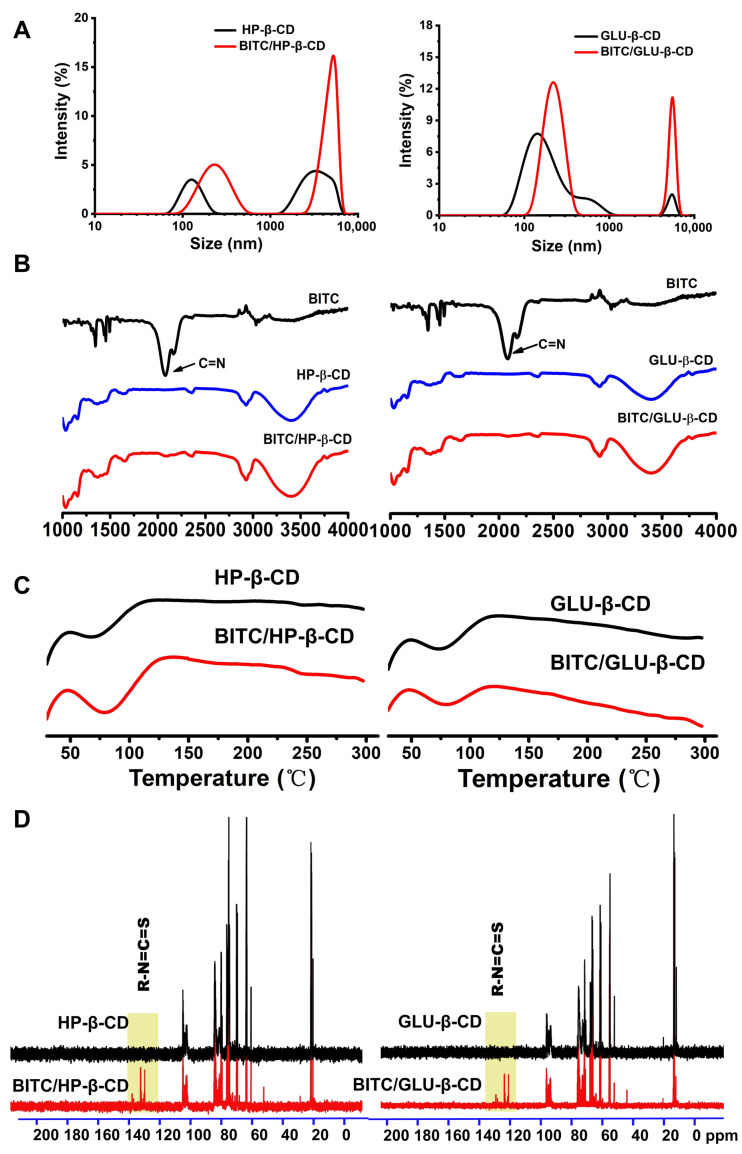
(**A**) Particle size of two β-cyclodextrin derivatives, including BITC. (**B**) IR spectra of free BITC, β-CD, and BITC inclusion complexes. (**C**) DSC spectra of β-CD and inclusion complexes. (**D**) ^1^H-NMR spectra of β-CD and inclusion complexes.

Particle sizes of HP-β-CD, GLU-β-CD, and their inclusion complexes were detected in triplicate. As shown in Figure 2A, the particle size distributions of HP-β-CD, GLU-β-CD, and BITC inclusion complexes all had two peaks. It could be seen from the figure that the particle size distribution of the β-CD derivative inclusion complex was similar to that of the pure β-CD derivative. The particle size results showed that the BITC in the mixture promoted the aggregation of β-CDs, thereby increasing the particle size of the solution to about 600 nm. This may be due to the hydrophobic interaction of BITC, which makes cyclodextrin easy to aggregate into large-size aggregates. From these differences, we may draw a conclusion that portions of BITC were embedded into the cyclodextrin hydrophobic cavity, causing these changes.

### 3.5. FT-IR Spectral Studies

FT-IR spectra of the raw BITC solution, cyclodextrin, and inclusion complexes were acquired to investigate any intermolecular interaction that could occur among various components and assess the formation of molecular complexes. The infrared spectra of BITC-β-CD complexes were similar to that of β-CDs (Figure 2B), indicating that encapsulation of BITC did not affect the molecular structures of β-CDs. In addition, the characteristic absorption peak of BITC disappeared at about 2250 cm^−1^ (C=N), indicating successful embedding of BITC in the β-CD cavities.

### 3.6. Thermodynamics Analysis

A broad and low endothermic peak was observed at 80–90 °C, which could be attributed to the liberation of crystal water from the β-CD cavities. The DSC graph of the BITC-β-CD complex showed the same thermodynamic characteristics as that of the original β-CDs (Figure 2C), which indicated that there was no interaction between BITC and the β-CDs in the physical mixtures. The results showed that BITC was dispersed in the cavities of the β-CD derivatives in a complex form without affecting the intrinsic structural state of the β-CD derivatives [35], and formed non-covalent bonds in the hydrophobic cavity of the latter. 

### 3.7. ^1^H-NMR Analysis

NMR was used to analyze the interaction between two molecules based on their chemical shift [36]. The ^1^H-NMR spectra of HP-β-CD, GLU-β-CD, and their inclusion complex with BITC are shown in Figure 2D. The change of the BITC-complexed HP-β-CD internal proton H-6 at 4 ppm was indicative of BITC embedding in the cavity. In contrast, the other exterior protons did not show any significant chemical change compared to plain CD, which indicated that the fundamental structure of HP-β-CD was unaltered after BITC embedding. The NMR result of GLU-β-CD was similar to that of HP-β-CD.

### 3.8. Anti-Bacterial and Anti-Tumor Activity

BITC is a compound that occurs naturally in cruciferous vegetables and is an effector molecule of the defense system of many cruciferous plants, such as broccoli. BITC mostly appears in masked forms known as glucosinolates (GLs) [37]. It is known that GLs exert their beneficial properties on human health after regular consumption in the diet of the edible parts of brassica vegetables rich in GLs. Additionally, Fahey et al. found that isothiocyanate (ITC) sulforaphane extracted from cruciferous plants such as broccoli has a strong bactericidal effect on Helicobacter pylori (including antibiotic-resistant strains) [38]. Yanaka et al. showed that sulforaphane not only enhanced the protective and reparative effects of gastric mucosa against oxidative stress, but also had anti-inflammatory effects on *H. pylori*-infected gastric mucosa in mice and humans [39]. In this study, we added BITC as a natural antibacterial drug to broccoli juice, which might enhance the antibacterial properties of broccoli. To control food spoilage, the use of small amounts of natural antimicrobial agents in food ingredients should be investigated in depth. However, the strong odor of BITC also limited its practical application. Research showed that the composite film based on chitosan incorporated with BITC and α-CD was able to effectively prolong the storage life of raw beef samples while retaining its sensory characteristics [40]. 

**Figure 3:**
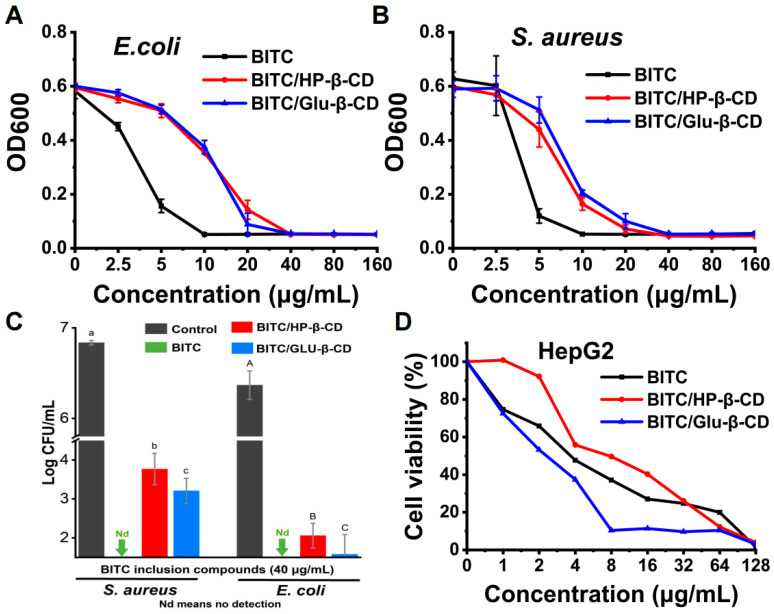
Anti-bacterial and anti-tumor activity of BITC-β-CDs. (**A**) and (**B**) Anti-bacterial activity of BITC, BITC/HP-β-CD, and BITC/GLU-β-CD on *E. coli* and *S. aureus*, respectively. (**C**) Bactericidal activity of BITC and BITC inclusion complexes against *S. aureus* and *E. coli*. Letters A–C and a–c represent statistically significant values at *p* ≤ 0.05. (**D**) Anti-tumor activity of BITC-β-CDs on HepG2.

The BITC inclusion complexes significantly inhibited the growth of *E. coli* (Figure 3A) and *S. aureus* (Figure 3B). The effective inhibitory concentration of HP-β-CD and GLU-β-CD against both strains was 20 μg/mL, whereas 40 μg/mL completely inhibited bacterial growth in liquid and solid nutrient medium. There was no significant difference in the antibacterial effect of BITC after inclusion with two β-CD derivatives. Combined with the previous experimental results of inclusion rates of the two β-CD derivatives, the higher inclusion rate GLU-β-CD was a better choice than HP-β-CD for inclusion BITC to form complexes. 

Figure 3C showed the plate colony-counting results of the experimental groups against *S. aureus* and *E. coli*. It can be clearly seen that the experimental groups exhibited antibacterial activities against both bacteria compared to the control group. BITC had the strongest antibacterial activity of these three groups, with 99.99% inhibition against *S. aureus* and *E. coli*, whereas BITC/HP-β-CD had the weakest antibacterial activity against *S. aureus* and *E. coli*.

Furthermore, HepG2 cells were treated with different concentrations of free BITC, BITC/HP-β-CD, and BITC/GLU-β-CD (0, 1, 2, 4, 8, 16, 32, 64, and 128 μg/mL). All three compounds exhibited inhibitory effects on the growth of HepG2 cells. Compared to free BITC, BITC/HP-β-CD decreased cytotoxicity, while BITC/GLU-β-CD increased cytotoxicity (Figure 3D). Additionally, 8 μg/mL of BITC/GLU-β-CD significantly inhibited the growth of HepG2 cells. This could be due to the higher solubility, permeability, and bioavailability of BITC/GLU-β-CD compared to free BITC and BITC/HP-β-CD, which enhanced BITC absorption and increased its inhibitory effect. 

### 3.9. Antimicrobial Stability of BITC-β-CDs in Broccoli Juice

**Figure 4:**
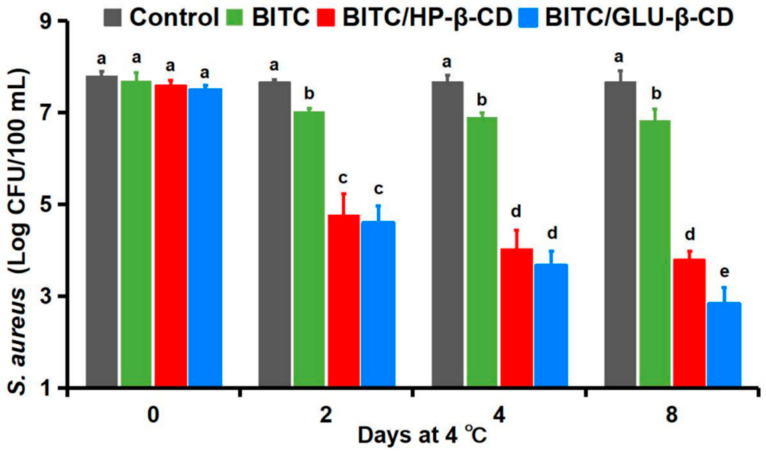
Antimicrobial activity of free and encapsulated BITC against *S. aureus* inoculated after pasteurization (63 °C, 30 min) of broccoli juice stored at 4 °C. Different letters among bars indicate significant differences among BITC treatments during storage time (*p* < 0.05).

Broccoli is more susceptible to contamination by *S. aureus* compared to tomatoes, mashed carrots, bok choy, cucumbers, and other vegetables [41]. In this study, broccoli juice samples inoculated with *S. aureus* were treated with free BITC, BITC/GLU-β-CD, and BITC/HP-β-CD for 0, 2, 4, and 8 days. The viability of the bacterial cells was greater in the samples treated with free BITC compared to the BITC/GLU-β-CD and BITC/HP-β-CD groups (Figure 4). However, the anti-bacterial effects of the different formulations gradually decreased with prolonged storage. Interestingly, the inhibitory effect of BITC/GLU-β-CD was significantly stronger than that of BITC/HP-β-CD at all time points within 8 days. This can be attributed to the embedding rate of BITC in GLU-β-CD being higher than that in HP-β-CD. 

Taken together, BITC/GLU-β-CD exhibited the optimum bacteriostatic effect against *S. aureus*. Cyclodextrin-coated BITC has good solubility and better flavor, which makes it suitable for use in broccoli juice. Our study provides a basis for the development of effective functional products (e.g., beverages) to extend the shelf life of foods and provides strong evidence that BITC and BITC-β-CD complexes may be effective in controlling *S. aureus* and *E. coli* strains in vegetable juices. In addition, the results of preserving broccoli juice with BITC-β-CD confirmed the potential bioefficacy of BITC, and it also opened new perspectives for the use of natural plant extracts as an opportunity to avoid synthetic chemical preservatives.

## 4. Conclusions

The present study showed the successful embedding of BITC in β-CD, overcoming the lacunas caused by the high volatility of BITC, increasing its solubility and bioactivity and reducing its toxicity. Molecule docking confirmed the formation of inclusion complexes between HP-β-CD, GLU-β-CD, and BITC, with the latter embedded into the CD cavity. The minimum inhibitory concentration (MIC) results showed that the BITC inclusion complexes exhibited better antimicrobial efficiency compared to pure BITC in both Gram-positive and -negative strains. The results of particle size, FT-IR, DSC, and ^1^H-NMR analyses also revealed that BITC was able to successfully form inclusion complexes. The results showed that the inclusion rate of GLU-β-CD was higher than that of HP-β-CD, while the inclusion stability of HP-β-CD was better than that of GLU-β-CD. BITC/GLU-β-CD can inhibit the growth of HepG2 cells in vitro. BITC-HP-β-CD and BITC-GLU-β-CD effectively inhibited the growth of both *E. coli* and *S. aureus*, and was able to prevent the growth of *S. aureus* in broccoli juice stored at 4 °C. These results indicate that BITC-β-CD complex has good application potential in the food industry.

## Data Availability

Data is contained within the article.
